# Effect of dialysate bicarbonate on calciprotein particle crystallization time (T50) in hemodialysis patients—the D-Bic study

**DOI:** 10.1093/ckj/sfaf263

**Published:** 2025-08-13

**Authors:** Jennifer Machacek, Peter S Neufeld, Andreas Pasch, Martina Gaggl, Maria C Haller, Edward R Smith, Daniel Cejka

**Affiliations:** Department of Medicine III – Nephrology, Hypertension, Transplantation, Rheumatology, Geriatrics, Ordensklinikum Linz – Elisabethinen Hospital, Linz, Austria; Department of Medicine III – Nephrology, Hypertension, Transplantation, Rheumatology, Geriatrics, Ordensklinikum Linz – Elisabethinen Hospital, Linz, Austria; Institute for Physiology and Pathophysiology, Johannes Kepler University Linz, Austria; Calciscon AG, Biel, Switzerland; Center for Public Health, Public Health Nutrition, Medical University Vienna, Vienna, Austria; Department of Medicine III – Nephrology, Hypertension, Transplantation, Rheumatology, Geriatrics, Ordensklinikum Linz – Elisabethinen Hospital, Linz, Austria; CeMSIIS – Center for Medical Statistics, Informatics, and Intelligent Systems, Medical University Vienna, Vienna, Austria; SEHA Kidney Care (SKC) Abu Dhabi, UAE; Department of Medicine III – Nephrology, Hypertension, Transplantation, Rheumatology, Geriatrics, Ordensklinikum Linz – Elisabethinen Hospital, Linz, Austria

**Keywords:** bicarbonate, calcification propensity, calciprotein particle, dialysis, T50

## Abstract

**Background:**

Short calciprotein crystallization time (low T50) is directly associated with an increased risk of cardiovascular events and mortality. Here, we investigated whether increases in dialysate bicarbonate concentrations increase T50 times in dialysis patients.

**Methods:**

In a prospective, single-center, single-arm, interventional trial in hemodialysis patients (*N* = 29), dialysate bicarbonate was decreased from baseline settings to 27 mmol/L (D-Bic 27) followed by an increase to 37 mmol/L (D-Bic 37), over the course of 6 weeks. The primary endpoint was the change in T50 time between the D-Bic 27 and D-Bic 37 phases. Measurements of endogenous calciprotein monomers (CPM), primary (CPP-1) and secondary (CPP-2) calciprotein particles were pre-specified secondary outcomes.

**Results:**

Twenty-four patients completed the study per protocol. T50 time increased significantly from 246 ± 77 to 282 ± 81 min from the D-Bic 27 to the D-Bic 37 phase (*P* < .0001). The hydrodynamic radius (size) of secondary calciprotein particles generated in the T50 test (CPP-2_Rh_) did not differ significantly between study phases (251 ± 75 vs 240 ± 78 nm, *P* = .27). Comparing the D-Bic 27 with the D-Bic 37 phase, CPM (16.8 × 10³ vs 16.2 × 10³ AU/µL, *P* = .9) and CPP-1 (4.6 × 105 vs 4.5 × 10^5^ counts/mL, *P* = .7) did not change significantly, but there was a significant decrease in CPP-2 levels (5.9 × 10^4^ vs 3.2 × 10^4^ counts/mL, *P* < .0003). Intradialytically, T50 increased, CPM and CPP-1 decreased, while CPP-2 remained stable.

**Conclusions:**

Raising dialysate bicarbonate resulted in a significant increase in T50 time and a reduction of CPP-2 levels.

## Graphical Abstract

**Graphical Abstract:**
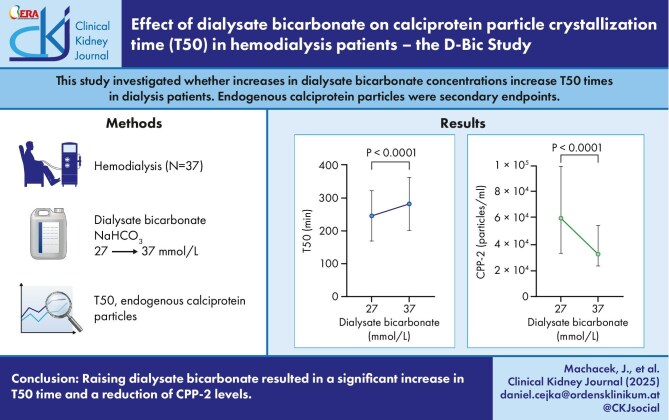

KEY LEARNING POINTS **What was known:**

- A short calciprotein particle crystallization time (low T50) is associated with cardiovascular events and mortality in dialysis patients.
- Serum bicarbonate levels associate positively with T50 times.
- Oral sodium bicarbonate supplementation did not increase T50 times in previous studies in non-dialysis chronic kidney disease patients.

**This study adds:**

- Proof-of-principle: increasing dialysate bicarbonate increases T50 times.

**Potential impact:**

- Modifying dialysate bicarbonate concentration might be a simple, readily available and inexpensive intervention to potentially improve dialysis patient outcomes in future studies.

## INTRODUCTION

Abnormalities in mineral metabolism, commonly summarized as chronic kidney disease–mineral and bone disorder (CKD-MBD) syndrome [1], are thought to be major drivers of the very high risk for cardiovascular events and death in patients with CKD, especially dialysis patients [2, 3]. The hepatic protein fetuin-A plays a major role in preventing ectopic crystallization of calcium–phosphate by chaperoning excess calcium phosphate into calciprotein particles (CPPs) [4]. High levels of CPPs are considered major contributors to the vascular damage found in CKD-MBD [5].

Measurement of serum calciprotein particle crystallization time *ex vivo* (T50 test) integrates the effect of several promoters (e.g. calcium, phosphate) and inhibitors (e.g. fetuin-A, magnesium, bicarbonate) of vascular damage into a single readout [6]. Low T50 times have been associated with cardiovascular morbidity and mortality in a broad variety of cohorts, such as the general population [7], patients with ischemic vascular diseases [8, 9], patients with CKD [10, 11], kidney transplant recipients [12, 13] and dialysis patients [14–16]. Therefore, various interventions to increase T50 times have been investigated during recent years [17–25], with the ultimate goal of improving patient outcomes in the future.

Serum bicarbonate levels have been repeatedly positively associated with T50 times in observational studies [13, 14, 16, 26]. However, interventional studies in non-dialysis-dependent CKD did not show an effect of oral bicarbonate supplementation on T50 times [27, 28].

Therefore, this study aimed to investigate whether an increase in dialysate bicarbonate concentrations increases T50 times.

## MATERIALS AND METHODS

### Patients

Patients were recruited at the Ordensklinikum Linz Elisabethinen dialysis facility, a tertiary care nephrological center. The main inclusion criteria were age ≥18 years and chronic (≥3 months) dialysis treatment with thrice weekly hemodialysis or hemodiafiltration (post-dilution, auto-mode for convection volume). Main exclusion criteria were severe chronic obstructive pulmonary disease (stage III or IV), history of severe hypercapnia or hypoxemia, and history of severe hypokalemia (<3.0 mmol/L) or hyperkalemia (>6.5 mmol/L) within 3 months prior to study inclusion. The first patient was recruited on 20 October 2023. The last patient visit was on 21 December 2023.

### Intervention

This a prospective, single-center, single-arm, interventional trial. All patients were treated using standard dialysis machines [model 6008^®^, Fresenius Medical Care (FMC), Bad Homburg, Germany] and high flux polysulfone dialyzers (FX series with Helixone^®^ membrane, FMC) with a centrally prepared dialysate (Granumix plus^®^, FMC) containing 3 mmol/L acetate, 0.5 mmol/L magnesium and 1.25 mmol/L calcium throughout the study. No other acids (e.g. citric acid or hydrochloric acid) were added. Bicarbonate was added on-line to the dialysis bath using a sodium bicarbonate dry concentrate (bibag^®^, FMC). The dialysate bicarbonate prescription was decreased from standard of care (mostly 35 mmol/L) in a stepwise fashion (2 mmol/L per change) to 27 mmol/L (D-Bic 27), and subsequentially increased again in a stepwise fashion (2 mmol/L per change) to 37 mmol/L (D-Bic 37) over the course of 6 weeks. Enoxaparin at a standard dose of 4000 IU (dose modified as clinically needed) was administered at the beginning of every dialysis session as intradialytic anticoagulant. Dialysis dose (Kt/V) was monitored at every dialysis session using an online clearance monitoring system (OCM^®^, FMC) without consideration of residual kidney function, and remained stable throughout the study. Concomitant medications, especially oral phosphate binders, calcimimetics and vitamin D preparations, as well as all other components of the dialysis bath, were held constant throughout the study as far as clinically justifiable as determined by the attending physicians. Blood was drawn immediately before initiation of hemodialysis via the dialysis access (arterio-venous fistula or central venous catheter). In patients who had a central venous catheter as dialysis access, 10 mL of blood were drawn and discarded prior to blood sampling for study purposes. To determine the intradialytic change of parameters of interest, one additional blood drawing was performed at the end of the dialysis sessions of the mid-week dialysis session of the D-Bic 27 as well as the D-Bic 37 phase, and patients were asked to remain fasted for these two dialysis sessions.

### Laboratory measurements

Blood was collected in gel separator tubes (SST, BD Vacutainer, Becton Dickinson, Plymouth, UK) for serum and K_3_EDTA tubes (Vacuette, Greiner BioOne, Kremsmünster, Austria) for plasma. Samples were centrifuged at 1280 × *g* for 15 min at room temperature and aliquots were transferred directly to –80°C for long-term storage.

Calciprotein crystallization time was measured in serum using the T50 test as described previously [14] at Calciscon (Calciscon AG, Biel, Switzerland) without knowledge of study phase. The hydrodynamic radius (Rh) of secondary calciprotein particles (CPP-2_Rh_) generated in the T50 test at 600 min, which has been associated with mortality in dialysis patients [29], was measured using 3D-dynamic light scattering as described previously [14, 30].

CPM [31, 32], CPP-1 and CPP-2 [33, 34] were analyzed as described previously, and assays are described in detail in the Supplementary data.

Routine blood tests were performed in the central laboratory facility of the Ordensklinikum Linz Elisabethinen hospital using Cobas analyzer systems (Roche Diagnostics, Rotkreuz, Switzerland). Blood gas analysis was performed using the RAPIDpoint 500 point-of-care blood gas system (Siemens Healthcare Diagnostics, Vienna, Austria). Intact parathyroid hormone (PTH) was measured using the Elecsys PTH (1-84) assay on a Cobas system (Roche). Intact fibroblast growth factor 23 (iFGF23) was measured in serum by enzyme-linked immunosorbent assay (Kainos, Tokyo, Japan).

### Sample size calculation and statistical methods

#### Sample size calculation

As changes in T50 in response to changes in dialysate bicarbonate prescription in dialysis patients were unknown, sample size calculation was based on estimations from previous studies [6, 10, 14, 26, 28] and the following assumptions: baseline mean T50 time: 243 min; increase in T50 time per mmol/L of increase in dialysate bicarbonate prescription: 4.6 min (i.e. 46 min per change of 10 mmol/L in dialysate bicarbonate prescription). Standard deviation (SD) of differences in T50 between low (27 mmol/L) and high (37 mmol/L) dialysate bicarbonate prescriptions was expected to be 63 min. Using a paired *t*-test, a sample size of *N* = 17 patients was calculated to achieve a statistical power of 0.8 with a two-sided significance level of .05. The sample size was calculated using nQuery Advisor software (Statistical Solutions, Cork, Ireland). To account for potential dropouts, which are typically frequent in a dialysis population due to the high burden of disease, the number of patients was increased by 40%, yielding an estimated sample size of 24 patients. Due to parallel recruitment, 29 patients were eventually recruited for this study, of which 26 patients were available at baseline, and 24 patients completed the study according to protocol.

#### Statistics

The characteristics of patients at study baseline are described by mean ± SD, median and interquartile range or frequency and percentage for normally distributed variables, non-normally distributed variables and categorical variables, respectively. The mean values of both study visits (i.e. after a long and short dialysis interval) during a given study phase were calculated for all laboratory parameters, including T50, to decrease intra-individual variation, which may result from day-to-day changes in diet in this outpatient setting. The primary endpoint, the change in T50 between low (D-Bic 27) and high (D-Bic 37) dialysate bicarbonate prescription, was tested for statistical significance using a paired *t*-test. The primary endpoint was investigated per-protocol as a pre-specified primary analysis. The per-protocol approach was chosen because this is a proof-of-principle study investigating the pharmacodynamic effects of dialysate bicarbonate on T50 while an intention-to-treat analysis ignores non-compliance, protocol deviations, withdrawal or similar, and therefore is not the first choice for the analysis of proof-of-principle studies [35]. Pre-specified secondary endpoints were changes in endogenous calciprotein particle levels between the D-Bic 27 and D-Bic 37 phases, as well as intradialytic changes of CPP levels during the mid-week dialysis session of the D-Bic 27 and D-Bic 37 phase, which were studied according to the distribution of data using a paired *t*-test or the Wilcoxon rank-sum test, respectively. Exploratory analyses comparing parameters at baseline and during low (D-Bic 27) and high (D-Bic 37) study phases were performed using repeated measures ANOVA followed by Tukey's *post hoc* test for multiple comparisons.

### Ethics

This study was conducted according to the International Conference on Harmonization on Good Clinical Practice (ICH-GCP) regulations and the Declaration of Helsinki on Ethical Principles for Medical Research Involving Human Subjects. Patients were only included in this study after providing oral and written informed consent. This study was reviewed and approved by the Ethics Committee of the Johannes Kepler University Linz (ID: 1140/2023) and prospectively registered with ClinicalTrials.gov (NCT06084858) on 10 October 2023.

## RESULTS

### Patients

Of 29 recruited patients, 26 patients were still available at study baseline, and 24 completed the study per protocol and thus were available for analysis of the primary endpoint. A CONSORT (Consolidated Standards of Reporting Trials) study flow diagram with further details and the dialysate bicarbonate prescriptions are shown in the Supplementary data (Supplementary data, Fig. S1 and Table S1). Patient demographics at baseline (*N* = 26) are shown in Table 1.

**Table 1:** Patient demographics of patients (*N* = 26) at baseline.

Age (years), median (IQR)	64 (61–76)
Sex, men/women, *n*/*n* (%/%)	16/10 (38/61)
Body mass index (kg/m²), mean (SD)	25.9 (5.2)
Primary renal disease, *n* (%)	
Diabetes	5 (19.2)
Hypertensive/vascular	6 (23)
ADPKD	3 (11.5)
Glomerulonephritis	0 (0)
Other	12 (46.2)
Dialysis vintage (months), median (IQR)	47 (28–66)
Previous kidney transplants, *n* (%)	
0	22 (84.6)
1	4 (15.4)
Vascular access, *n* (%)	
AV fistula	18 (69.2)
Catheter	8 (30.8)
Laboratory parameters baseline	
Calcium, albumin-corrected (mmol/L), mean (SD)	2.22 (0.11)
Phosphate (mmol/L), mean (SD)	1.83 (0.56)
Bicarbonate (mmol/L), mean (SD)	21.8 (2.8)
Magnesium (mmol/L), mean (SD)	1.10 (0.19)
iPTH (pg/dL), median (IQR)	249 (148–445)
Hemoglobin (g/dL), mean (SD)	11.6 (1.2)
Comorbidities, *n* (%)	
Diabetes mellitus	10 (61.5)
Coronary artery disease	12 (46.1)
History of myocardial infarction	4 (15.3)
History of congestive heart failure	8 (30.7)
Atrial fibrillation	2 (7.6)
Peripheral occlusive vascular disease	7 (26.9)
History of amputation	2 (7.6)
Cerebrovascular disease	4 (15.3)
History of stroke or TIA	1 (3.8)
Cigarette smoking	4 (15.3)
Dialysis treatment, *n* (%)	
HD	11 (42.3)
HDF	15 (57.7)
HDF convection volume achieved (L), mean (SD)	17.9 (3.69)
Duration per session (min), median (IQR)	240 (240–240)
Kt/V, mean (SD)	1.15 (0.25)
Blood flow achieved (mL/min), median (IQR)	288 (249–297)

AV fistula, arterio-venous fistula; iPTH, intact PTH; TIA, transitory ischemic attack; HD, hemodialysis; HDF, hemodiafiltration; Kt/V, dialysis dose, measured with online clearance monitoring (OCM) of plasma sodium; SD, standard deviation; IQR, interquartile range.

### Calciprotein particle crystallization time (T50) and endogenous CPP levels

**Figure 1:**
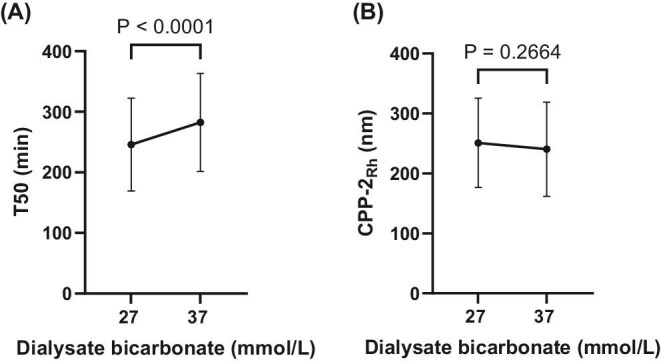
Serum calciprotein crystallization time (T50 time; (**A**) and secondary calciprotein size (CPP-2_Rh_; (**B**) during D-Bic 27 and D-Bic 37 study phases.

The T50 time increased significantly from 246 ± 77 to 282 ± 81 min from the D-Bic 27 to the D-Bic 37 phase (*P* < .0001, Fig. 1A). The Rh (size) of secondary calciprotein particles generated in the T50 test (CPP-2_Rh_) did not differ significantly between the D-Bic 27 and D-Bic 37 phase (251 ± 75 vs 240 ± 78 nm, *P* = .27, Fig. 1B).

**Figure 2:**
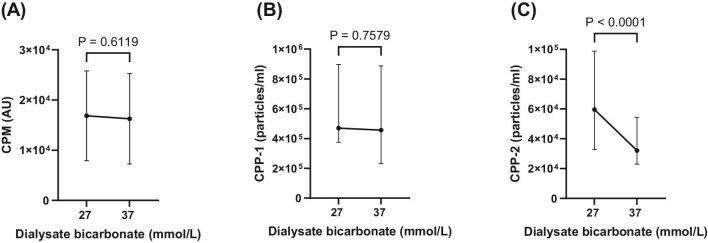
Endogenous levels of CPM (arbitrary units; (**A**), primary (CPP-1; (**B**) and secondary (CPP-2; (**C**) CPPs during D-Bic 27 and D-Bic 37 study phases.

Comparing the D-Bic 27 with the D-Bic 37 phase, neither CPM (16.8 ± 8.9 × 10³ vs 16.2 ± 8.9 × 10³ AU/µL, *P* = .9) nor CPP-1 [4.6 (3.7 to 8.9) × 10^5^ vs 4.5 (2.3 to 8.8) × 10^5^, *P* = .7] counts/mL changed significantly, but there was a significant decrease in CPP-2 levels from 5.9 (3.2 to 9.8) × 10^4^ to 3.2 (2.3 to 5.4) × 10^4^ counts/mL (*P* < .0003). Results are shown in Fig. 2A–C.

Results of exploratory longitudinal analyses, including baseline values, are shown in Supplementary data, Fig. S2A–D.

### Other laboratory parameters

**Figure 3:**
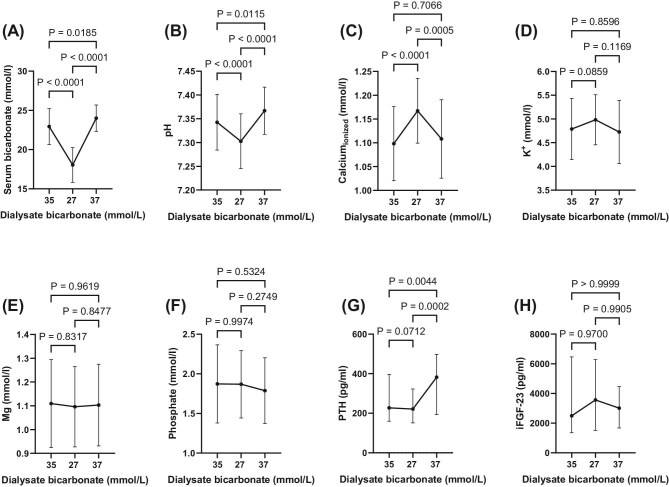
Longitudinal measurements of serum bicarbonate (**A**), pH (**B**), ionized calcium (**C**), potassium (**D**), magnesium (**E**), phosphate (**F**), PTH (**G**) and iFGF23 (**H**).

Results of exploratory analyses of other laboratory measurements are shown in Fig. 3A–H. Comparing the D-Bic 27 to the D-Bic 37 phase, serum bicarbonate increased from 18.0 ± 2.2 to 24.0 ± 1.6 mmol/L (*P* < .0001), serum pH increased from 7.30 ± 0.05 to 7.36 ± 0.04 (*P* < .0001), while serum ionized calcium decreased from 1.16 ± 0.06 to 1.10 ± 0.08 mmol/L (*P* < .0005). Interestingly, serum potassium remained relatively stable throughout the study, although the dialysis potassium prescription was changed (at the discretion of the treating physicians) only five times in three patients. Serum phosphate, magnesium and FGF23 remained relatively stable throughout the study, while PTH increased significantly from the D-Bic 27 to the D-Bic 37 phase, from 221 (151 to 322) to 382 (193 to 497) pg/mL (*P* < .0002).

### Intradialytic change of T50 and CPPs

**Figure 4:**
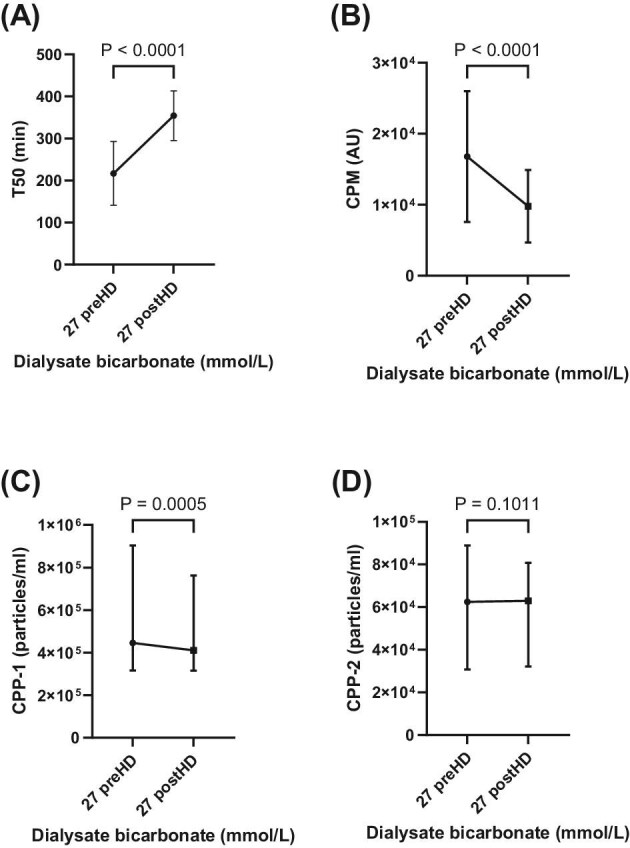
Intradialytic changes of calciprotein particle crystallization time (T50; (**A**)) and endogenous levels of CPM (arbitrary units; (**B**), primary (CPP-1; (**C**) and secondary (CPP-2; (**D**) CPPs at a dialysate bicarabonate prescription of 27 mmol/L. preHD, before dialysis; postHD, after dialysis.

Regarding intradialytic changes, in the D-Bic 27 phase, T50 increased significantly (from 217 ± 75 to 354 ± 58 min, *P* < .0001), while CPM (from 16.7 × 10³ ± 9.2 to 9.7 10³ ± 5.1 AU/µL, *P* < .0001) and CPP-1 [from 4.4 (3.1 to 9.0) × 10^5^ vs 4.1 (3.1 to 7.6) × 10^5^ counts/mL, *P* < .0005] decreased significantly, whereas CPP-2 [from 6.2 (3.0 to 8.8) × 10^4^ to 6.2 (3.2 to 8.0) × 10^4^ counts/mL, *P* = .10] did not change significantly (Fig. 4). The magnitude of these intradialytic changes was similar in the D-Bic 37 phase (T50: from 273 ± 74 to 404 ± 96 min, *P* < .0001; CPM: from 16.6 ± 9.7 × 10³ to 8.0 ± 5.1 × 10³ AU/µL, *P* < .0001; CPP-1: from 4.7 (2.1 to 10.2) × 10^5^ vs 4.0 (1.9 to 7.6) × 10^5^ counts/mL, *P* < .0001; CPP-2: from 3.2 (1.6 to 5.1) × 10^4^ to 3.0 (1.6 to 5.8) × 10^4^ counts/mL, *P* = .14; Supplementary data, Fig. S3).

### Adverse events

Generally, the changes in serum bicarbonate prescription were well tolerated by patients. In particular, intradialytic muscle cramps prompting a change of treatment (e.g. decrease in ultrafiltration rate, administration of 5% glucose intravenous or similar measures), which might have been expected due to changes in ionized calcium levels with changes in serum pH due to changes in the dialysate bicarbonate prescription, did not occur at all during the duration of the study.

During the active phase of the study, 15 adverse events and 2 serious adverse events resulted in two study drop-outs, as expected in a dialysis population due to the high burden of disease. Reasons and timing for study drop-outs according to study phase are detailed in the CONSORT flow chart in the Supplementary data, Fig. S1. Adverse events occurred for various reasons, most of which were infection-related, such as COVID-19 or *Clostridium difficile* colitis. The two serious adverse events are described in detail in the Supplementary data.

## DISCUSSION

The primary finding of our study was that higher dialysate bicarbonate prescriptions resulted in longer T50 times. Specifically, each 1 mmol/L increase in dialysate bicarbonate was associated with a 3.6-min increase in T50. This observed increase closely matched our initial estimate of a 4.6-min rise in T50 per 1 mmol/L increase, which was used to determine the sample size. In contrast to our study, previous studies using oral sodium bicarbonate supplementation in patients with non-dialysis CKD did not find any influence of bicarbonate treatment on T50 times [27, 28]. The most likely explanations for this discrepancy are the relatively small effect size of serum bicarbonate levels on T50 values and the dose limitations of oral bicarbonate supplementation compared with parenteral supplementation with dialysate. In the studies by Kendrick *et al*. [27] and Aigner *et al*. [28], the differences in serum bicarbonate levels between the intervention and control groups were 2.4 and 0.6 mmol/L, respectively. Assuming an increase in T50 of approximately 5 min per increase in serum bicarbonate levels, the expected increases in T50 times would have been around 12 and 3 min, respectively. In combination with a typical standard deviation of T50 between 60 and 80 min, these studies were probably underpowered to detect an effect of oral bicarbonate supplementation on T50 times in patients with non-dialysis-dependent CKD. Even in our study, where we used a large increase in dialysate bicarbonate prescription, the increase in T50 was relatively modest when compared with other interventions, such as phosphate lowering by oral phosphate binder therapy [23] or increasing serum magnesium concentrations using magnesium-enriched dialysate [22]. Therefore, it seems unlikely that increases in dialysate bicarbonate prescription as a solitary intervention will lead to meaningful improvements in patient outcomes when studied in a phase 3 clinical trial. However, we envision increases in dialysate bicarbonate prescription to be part of a multi-interventional approach to maximize T50 times and investigate the benefits of improved T50 in future studies with patient-level outcomes.

The Rh (size) of secondary calciprotein particles generated in the T50 test (CPP-2_Rh_) did not differ significantly between the D-Bic 27 and D-Bic 37 phase. This finding is in line with our expectations, as CPP-2_Rh_ is mainly influenced by the protein content (e.g. fetuin A) of CPPs generated in the T50 test [36], not small molecules such as bicarbonate.

Another significant finding of our study was that higher dialysate bicarbonate prescriptions reduced pre-dialytic (trough) levels of endogenous CPP-2, while CPM and CPP-1 levels remained unchanged. This suggests that bicarbonate does not appreciably affect the formation of CPM or CPP-1 but instead slows the conversion of CPP-1 to CPP-2, a process characterized by the formation of crystalline calcium–phosphate (hydroxyapatite) crystals [4]. Assuming a constant scavenger receptor clearance of CPP-2 by hepatic Kupffer cells (as described previously [37]), this mechanism would explain lower levels of endogenous CPP-2 found under high dialysate bicarbonate conditions. Toxicities of CPP depend on cell type and *in vitro* conditions [38], but in particular CPP-2 have been reported to be important drivers of endothelial cell damage, apoptosis and vascular smooth muscle cell calcification [39, 40]. Although we did not perform a formal assessment of inter-dialytic kinetics, our data support the notion of decreased CPP-2 load with increased dialysate bicarbonate prescription. Thus, increasing dialysate bicarbonate prescription harbors the potential of reducing vascular damage by reducing exposure of vessels to CPP-2.

In contrast to pre-dialytic CPP levels and regardless of the dialysate bicarbonate prescription, CPM and CPP-1 decreased during dialysis, whereas CPP-2 levels remained unchanged. This observation aligns with the current understanding of calciprotein particle biology: CPM and CPP-1 consist of amorphous calcium–phosphate complexes that can dissolve readily when mineral concentrations shift during dialysis (for example, when serum phosphate falls intradialytically). In contrast, once calcium–phosphate matures into crystalline hydroxyapatite as found in CPP-2, its extremely low solubility renders them resistant to intradialytic dissolution. This mechanism may partly explain why dialysis patients, even when adequately dialyzed by current standards (i.e. Kt/V above recommended thresholds), still exhibit excessive cardiovascular risk. Moreover, because conventional hemodialysis cannot remove certain uremic toxins, simply increasing the dialysis dose (Kt/V) does not necessarily translate into improved outcomes [41]. Instead, removing CPP-2 by complimentary treatments to dialysis, such as the introduction of a CPP adsorption column into the dialysis circuit as recently demonstrated in a pre-clinical model of kidney failure [42], is a novel and promising approach to ultimately improve patient survival.

In our current study we used acetate as the acid component of dialysate. Other acids can be used instead of acetate, and especially citrate-buffered dialysate has gained clinical interest for a variety of potential benefits over acetate. However, in a previous clinical trial (CitMag study) we did not find an effect of substituting acetate for citrate in combination with increased dialysate magnesium concentration on the T50 time in dialysis patients, probably due to increased complexation of magnesium by citrate [43].

In the current study, PTH increased while ionized calcium decreased between the D-Bic 27 and 37 mmol/L phases, as would be expected based on our current understanding of PTH physiology. One might speculate that this increase in PTH leads to increases in CPP blood levels. Indeed, PTH is a major driver of bone cellular activity (particularly osteoclasts) and CPPs are thought to partly originate from bone [5], cessation of cinacalcet is associated with increased PTH and CPP levels [44], and lowering of PTH using etelcalcetide decreases CPP levels [24]. However, in our study serum levels of CPM and CPP-1 remained unchanged between the D-Bic 27 and 37 mmol/L phases, and CPP-2 even decreased. A possible explanation for this discrepancy is that changes in median PTH levels in the current study were considerably smaller in magnitude (∆PTH ≈ 161 pg/mL) compared with previous reports (∆PTH ≈ 684 pg/mL [44]; ∆PTH ≈ 430 pg/mL [24]). Therefore, the effect of changes in PTH on bone turnover may have been too subtle to be detectable in CPP levels in the current study.

This study has several limitations, the major one being the magnitude of change in dialysate bicarbonate prescription of 10 mmol/L used in this study. This was done under stringent control of serum electrolytes throughout the study and was necessary to provide proof-of-principle but certainly will not be feasible for daily clinical routine due to concerns of over-alkalinization. High serum bicarbonate levels as well as high dialysate bicarbonate prescriptions have been associated with adverse outcomes including increased patient mortality [45, 46]. While high serum bicarbonate levels may be more of a marker of malnutrition (due to lack of dietary acid load) and not a major concern *per se*, high dialysate bicarbonate prescriptions certainly can induce electrolyte disturbances (hypokalemia, low ionized calcium), which may be causal for episodes of arterial hypotension and cardiac arrhythmias [47]. The optimal dialysate bicarbonate prescription is still a matter of lively debate [48, 49]. From a physiological and clinical perspective, we strongly suggest that changes in dialysate bicarbonate prescription should be done gradually, accompanied by monitoring for changes in serum potassium and ionized calcium, and that over-alkalinization should be avoided. Still, many patients are being treated with dialysate bicarbonate prescriptions around 32 mmol/L [46], which may allow for slight increases in dialysate bicarbonate prescriptions in clinical routine, yielding a modest increase in T50 time. Further limitations are the open-label, single-arm, single-center study design. This limits the generalizability of our findings and is certainly not sufficient to advocate for a general change in dialysate bicarbonate prescription practice. Our trial was clearly intended to provide proof-of-concept only as a basis for future studies. Another limitation is that changes in dialysate bicarbonate prescription lead to both, changes in serum bicarbonate and serum pH. Therefore, it is difficult to distinguish in a clinical setting whether the effects of dialysate bicarbonate prescription on T50 are mediated by the bicarbonate molecule as such, or rather the pH. Possibly, this could be further elucidated by using *in vitro* studies with different weak (e.g. carbonic, acetic, citric, maleic) or strong (e.g. hydrochloric) acids. On theoretical grounds, one could argue that the effect of bicarbonate on T50 is mediated more by the bicarbonate molecule than by pH. The T50 test uses a high concentration (100 mM) of HEPES buffer, which adjusts the serum sample to a pH close to 7.4. Because in our clinical study increases in bicarbonate still led to increases in T50 under these strongly buffered conditions, this suggests that bicarbonate itself, not pH, drives the effect. In our study, an increase in dialysate bicarbonate concentration led to lower trough levels of endogenous CPP-2. Again, the question arises of whether this effect is mediated more by the changes in pH, or by bicarbonate itself. Although total serum phosphate remained stable throughout the study, raising the pH shifts the phosphate equilibrium (H₂PO₄⁻ ⇌ HPO₄²⁻) towards HPO₄²⁻ which is the pool of phosphate species more readily incorporated into calcium–phosphates. This may favor hydroxyapatite formation and thus higher endogenous CPP-2 levels. However, raising the dialysate bicarbonate from 27 to 37 mmol/L increased mean blood pH only from 7.30 to 7.36, a shift that is calculated to increase HPO₄²⁻ activity from 0.235 mM at pH 7.30 to 0.249 mM at pH 7.36, and therefore likely has minimal impact on calcium–phosphate supersaturation.

In contrast, bicarbonate can substantially inhibit calcium–phosphate crystallization through two principal mechanisms: first, it forms soluble Ca–HCO₃⁻ ion pairs that lower the activity of free Ca²⁺, reducing supersaturation with respect to amorphous calcium–phosphate. Second, at higher concentrations, carbonate (CO₃²⁻) derived from bicarbonate incorporates into the amorphous calcium–phosphate network, disrupting long-range phosphate ordering and raising the activation energy required for crystallization. These thermodynamic and structural effects of bicarbonate are predicted to outweigh any minor change in phosphate speciation.

The strengths of the study include the clear-cut study design and a well-defined, easily controlled study intervention without the need for patient adherence to study medication.

In conclusion, increases in dialysate bicarbonate prescription significantly increased T50 times and reduced endogenous CPP-2 levels.

## Supplementary Material

sfaf263_Supplemental_File

## Data Availability

Data may be shared with researchers upon reasonable request.
