# Supplementary material for: Effect of dialysate bicarbonate on calciprotein particle crystallization time (T50) in hemodialysis patients—the D-Bic study
Source: Clin Kidney J. 2025 Aug 13;18(9):sfaf263. doi: 10.1093/ckj/sfaf263 (PMC12421725; doi:10.1093/ckj/sfaf263)
Supplement: sfaf263_Supplemental_File [file sfaf263_supplemental_file.pdf]

Table S1A - Dialysate Bicarbonate prescription by dialysis day - Monday-Wednesday-Friday

Week of study	Day of study	Day of week	Study visit number	Dialysate bicarbonate prescription (mmol/l)	
1	1	Monday	V1	35	
	2	Tuesday			
	3	Wednesday	V2	33	
	4	Thursday			
	5	Friday		31	
	6	Saturday			
	7	***Sunday***			
2	8	Monday	V3	29	
	9	Tuesday			
	10	Wednesday		27	
	11	Thursday			
	12	Friday		27	
	13	Saturday			
	14	***Sunday***			
3	15	Monday	V4	27	
	16	Tuesday			
	17	Wednesday	V5 + postHD sampling	27	
	18	Thursday			
	19	Friday		29	
	20	Saturday			
	21	***Sunday***			
4	22	Monday	V6	31	
	23	Tuesday			
	24	Wednesday		33	
	25	Thursday			
	26	Friday		35	
	27	Saturday			
	28	***Sunday***			
5	29	Monday	V7	37	
	30	Tuesday			
	31	Wednesday		37	
	32	Thursday			
	33	Friday		37	
	34	Saturday			
	35	***Sunday***			
6	36	Monday	V8	37	
	37	Tuesday			
	38	Wednesday	V9 + postHD sampling	37	End of study
	39	Thursday			
	40	Friday		35	
	41	Saturday			
	42	***Sunday***			

Table S1B - Dialysate Bicarbonate prescription by dialysis day - Tuesday-Thursday-Friday

Week of study	Day of study	Day of week	Study visit number	Dialysate bicarbonate prescription (mmol/l)	
1	1	Monday			
	2	Tuesday	V1	35	
	3	Wednesday			
	4	Thursday	V2	33	
	5	Friday			
	6	Saturday		31	
	7	***Sunday***			
2	8	Monday			
	9	Tuesday	V3	29	
	10	Wednesday			
	11	Thursday		27	
	12	Friday			
	13	Saturday		27	
	14	***Sunday***			
3	15	Monday			
	16	Tuesday	V4	27	
	17	Wednesday			
	18	Thursday	V5 + postHD sampling	27	
	19	Friday			
	20	Saturday		29	
	21	***Sunday***			
4	22	Monday			
	23	Tuesday	V6	31	
	24	Wednesday			
	25	Thursday		33	
	26	Friday			
	27	Saturday		35	
	28	***Sunday***			
5	29	Monday			
	30	Tuesday	V7	37	
	31	Wednesday			
	32	Thursday		37	
	33	Friday			
	34	Saturday		37	
	35	***Sunday***			
6	36	Monday			
	37	Tuesday	V8	37	
	38	Wednesday			
	39	Thursday	V9 + postHD sampling	37	End of study
	40	Friday			
	41	Saturday		35	
	42	***Sunday***			

D-Bic-HD-T50 study - CONSORT Flow Diagram

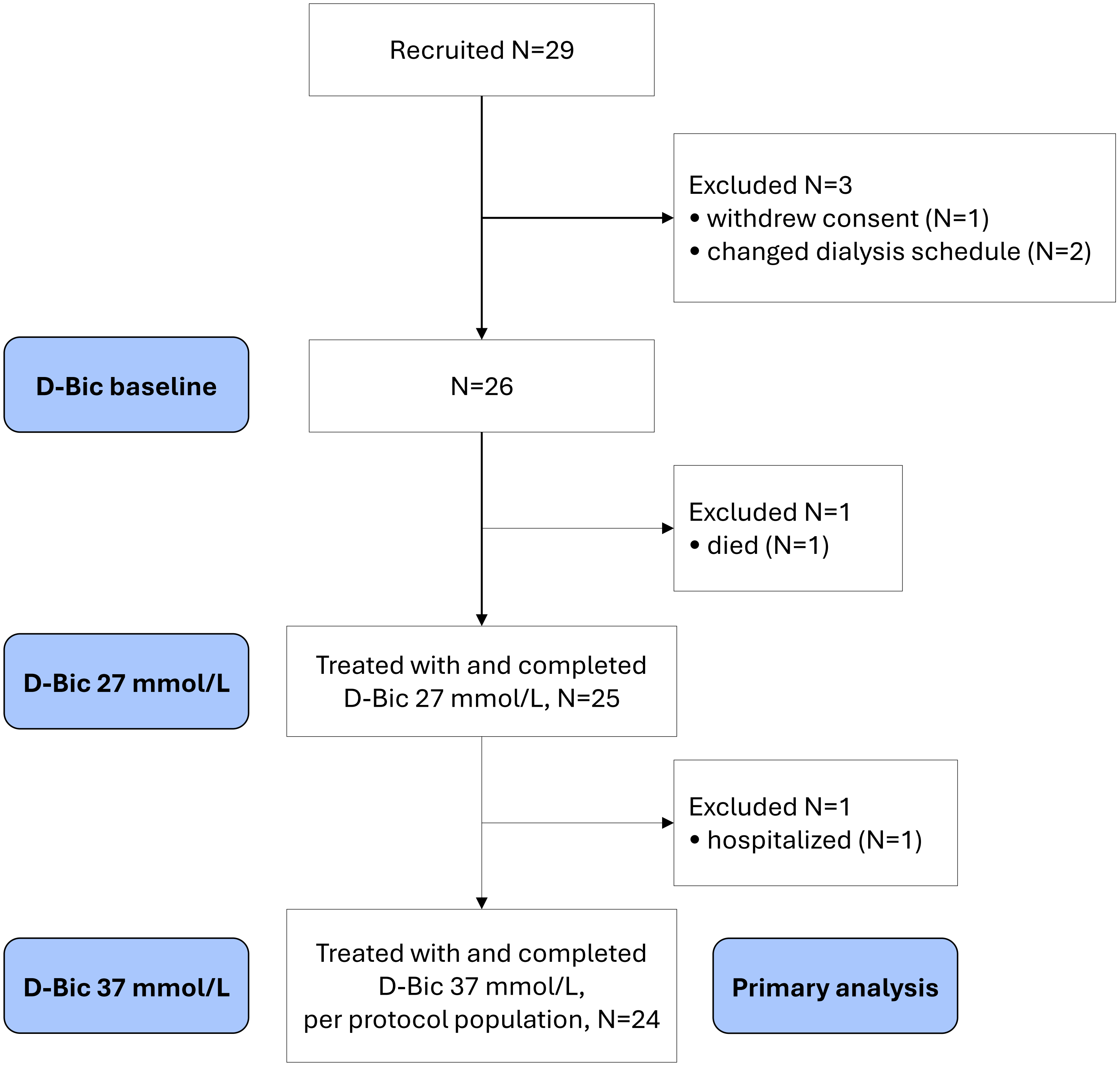

Supplementary Figure S1
CONSORT study flow diagram

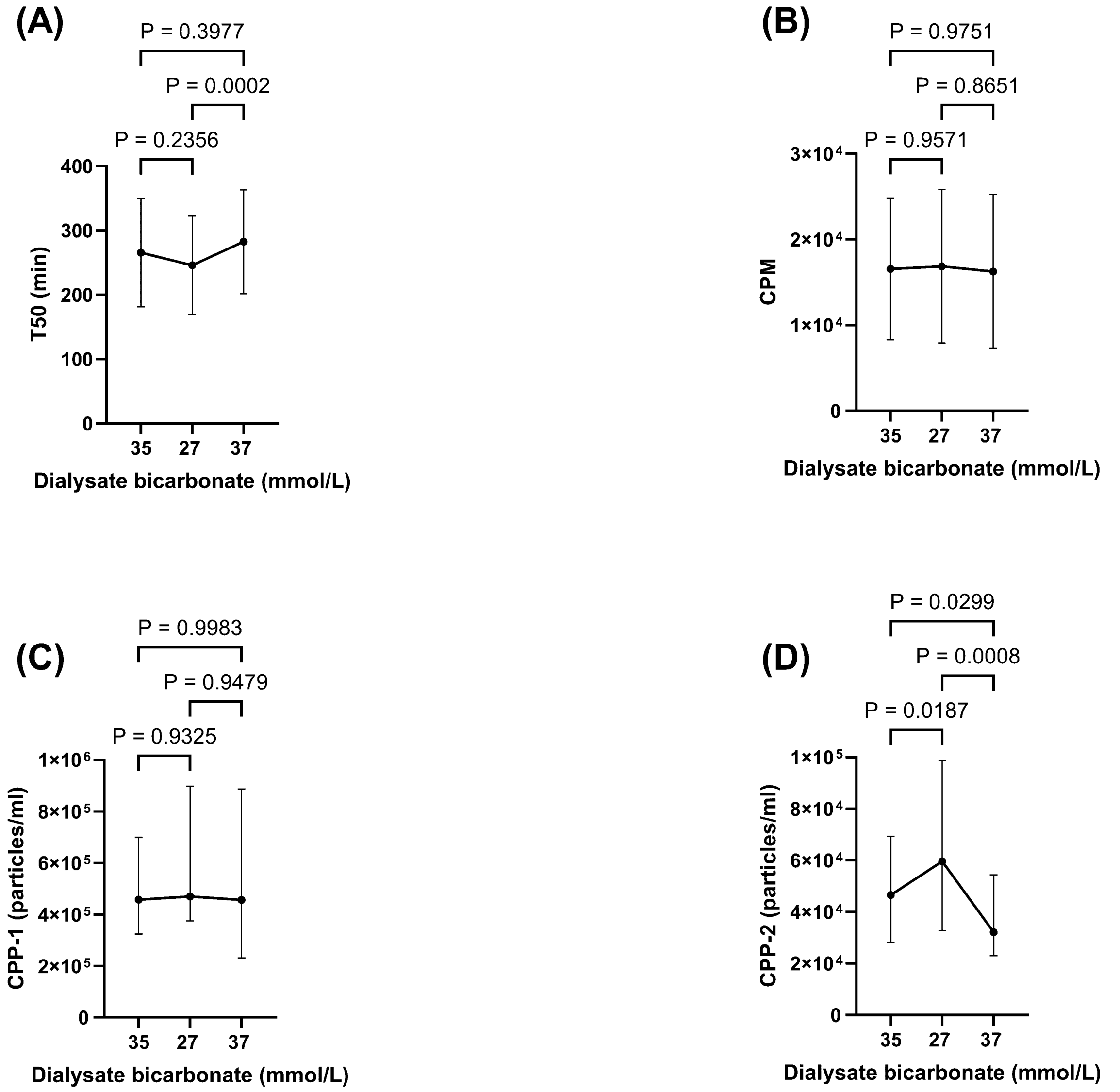

Supplementary Figure S2 – Longitudinal measurements of T50 and endogenous CPPs

Serum calciprotein crystallization time (T50 time; A), Endogenous levels of calciprotein monomers (CPM, arbitrary units; B), primary (CPP-1; C) and secondary (CPP-2; D) calciprotein particles at baseline (dialysate bicarbonate predominantly 35 mmol/L), the D-Bic 27 and D-Bic 37 study phases. Repeated measures ANOVA followed by Tukey's post hoc test.

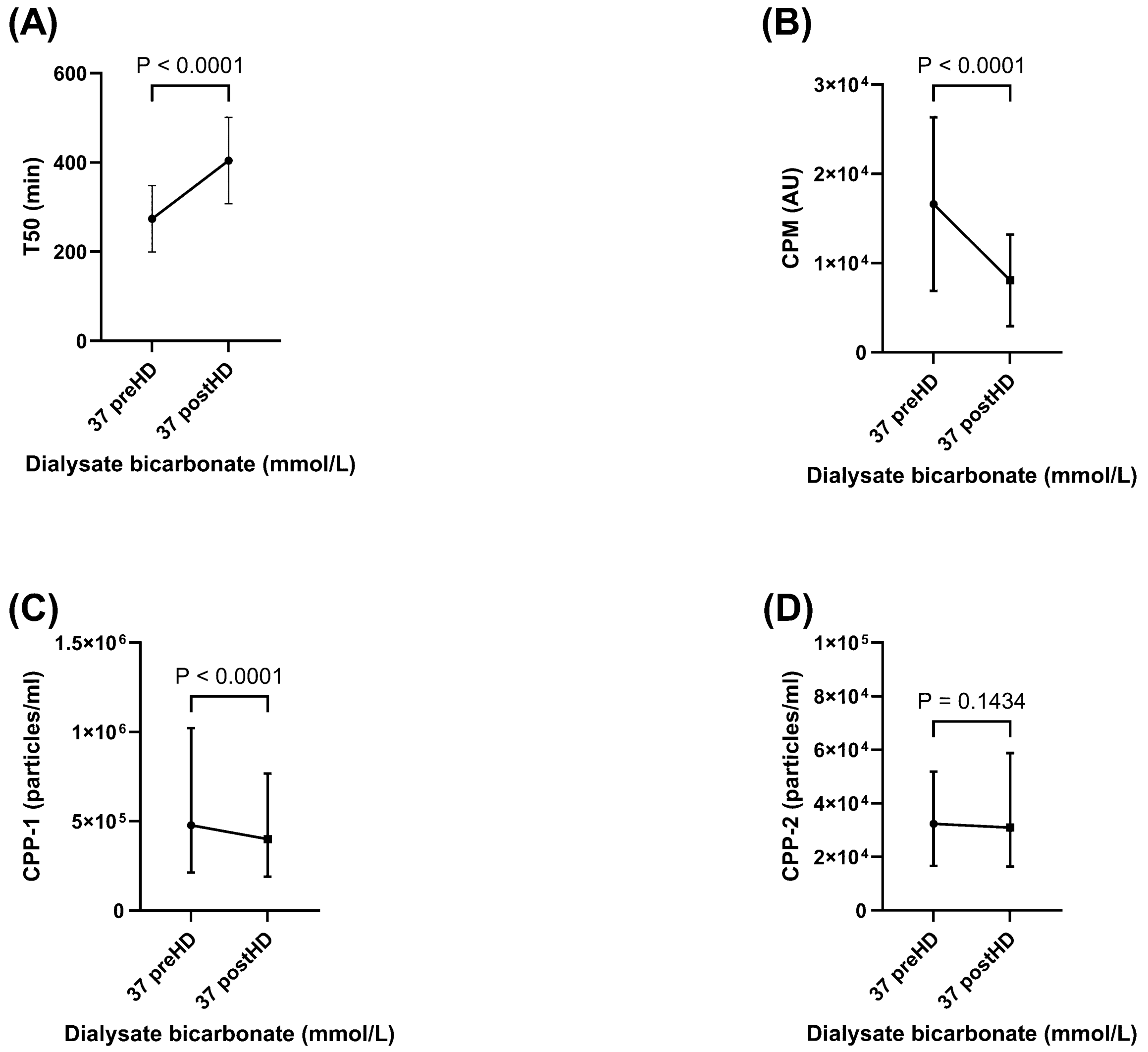

Supplementary Figure S3

Intra-dialytic changes of serum calciprotein crystallization time (T50 time; A), endogenous levels of calciprotein monomers (CPM, arbitrary units; B), primary (CPP-1; C) and secondary (CPP-2; D) calciprotein particles at a dialysate bicarbonate prescription of 37 mmol/L. Paired t-test or Wilcoxon rank-sum test, respectively.

Detailed description of serious adverse events

One patient, a 61-year-old male with a history of insulin-dependent diabetes mellitus type 2, ischemic coronary artery disease, aortic valve stenosis, parathyroidectomy due to refractory secondary hyperparathyroidism and recurrent calcifying uremic arteriolopathy (calciphylaxis) at the lower extremities, died during the study. On the day of his death, the patient reported feeling unwell with chest pain before the dialysis session. After exclusion of acute ST-segment elevation myocardial infarction (STEMI), dialysis treatment was initiated. Approximately 1 hour into the dialysis session, the patient developed progressive typical angina, followed by ventricular arrhythmia and subsequent cardiac arrest and died despite all resuscitation efforts. The last laboratory results drawn before dialysis on the day of his death showed deteriorating anemia (hemoglobin of 9.4 g/dl), glucose metabolism (glucose 311 mg/dl), inflammation (C-reactive protein of 6.4 mg/dl) and signs of myocardial ischemia (high sensitivity troponin T of 253 pg/ml) but electrolytes within normal range: serum potassium of 4.81 mmol/L (reference: 3.2 to 5.2 mmol/L) and a serum albumin-corrected calcium of 2.2 mmol/L (reference: 2.1 to 2.5 mmol/L). Therefore, this death was attributed to acute myocardial ischemia, possibly aggravated by an incipient infection, and not related to the study intervention. The other patient with a serious adverse event, a 63-year-old male with autosomal dominant polycystic kidney disease, a history of prior kidney transplantation with early graft failure, unsuccessful kidney transplantation due to hypotension, ischemic cardiomyopathy with an implantable cardioverter-defibrillator, peripheral artery disease with a history of bilateral amputation of the lower extremities and permanent atrial fibrillation treated with warfarin. He was hospitalized in another hospital due to basilar artery thrombosis, which was successfully treated by mechanical thrombectomy. Electrolytes were within normal limits at the last study visit 3 days before hospitalization: serum potassium of 4.95 mmol/L, and a serum albumin-corrected calcium of 2.2 mmol/L. The basilar artery thrombosis was attributed to a thromboembolic event due to atrial fibrillation and therefore not attributed to the study intervention by investigators.

Supplementary materials and methods

For CPM analysis, samples (25 µl) were centrifuged at 30,000 g and 4 °C for 2 hours to pellet CPP. The supernatants were incubated at 37 °C with gentle shaking for 24 hours. Subsequently, 5 µl of supernatant was mixed with 45 µl HEPES-buffered DMEM (100 mM HEPES, pH 7.8, no phenol red) containing 5 µM Alexa Fluor 647-risedronate (BioVinc, Pasadena, CA, US). After a 1-hour light-protected incubation at room temperature, the unbound dye was removed via gel filtration using pre-equilibrated Micro Bio-Spin Columns with Bio-Gel P-30 (Bio-Rad). The resulting 40 µl flow-through was combined with 10 µl of 0.5 M EDTA (pH 8.0) and 10 µl of 10% SDS in water. Samples were assayed in triplicate, and fluorescence was measured using a Synergy HTX multimode plate reader (BioTek, Winooski, VT, US)(1, 2).

For CPP-I and CPP-II analysis, samples (5 µl) were mixed with HEPES-buffered DMEM (40 µl: 50 mM HEPES, pH 7.45, no phenol red) and staining solution (5 µl) containing 5 µM Alexa Fluor 647-conjugated risedronate, 1.5 µg/ml FITC-conjugated lactadherin (Hematologic Technologies Inc., Essex Junction, VT, USA), and 2 µg/ml mFluor Violet 450-labelled bovine fetuin-A (prepared in-house using the ReadiLink Rapid mFluor Violet 450 Labeling Kit, AAT Bioquest, Pleasanton, CA, US). The mixture was incubated in the dark with gentle mixing for 120 minutes and then diluted to 500 µl with HEPES-buffered DMEM. Samples were analyzed in triplicate using an Apogee A50/Micro flow cytometer (Apogee Flow, Spain) equipped with 405, 488, and 638 nm lasers. Sheath pressure was set to 150 mbar, and four flush cycles were performed following each sample to reduce carryover. A 3 µl/min flow rate was used, and measurements were acquired for 120 seconds or until 5×10^6 events were recorded. Fetuin-A-positive and lactadherin-negative CPPs were detected using fluorescence thresholding. CPP-I and CPP-II were differentiated based on their affinity for risedronate, with risedronate_LO identifying CPP-I and risedronate_HI identifying CPP-II.(3, 4).

1. Miura Y, Iwazu Y, Shiizaki K, et al.; Identification and quantification of plasma calciprotein particles with distinct physical properties in patients with chronic kidney disease. Sci Rep 2018; 8(1):1256.

2. Tiong MK, Holt SG, Ford ML, Smith ER; Serum Calciprotein Monomers and Chronic Kidney Disease Progression. Am J Nephrol 2022; 53(11-12):806-815.
3. Smith ER, Hewitson TD, Cai MMX, et al.; A novel fluorescent probe-based flow cytometric assay for mineral-containing nanoparticles in serum. Sci Rep 2017; 7(1):5686.
4. Smith ER, Pan FFM, Hewitson TD, Toussaint ND, Holt SG; Effect of Sevelamer on Calciprotein Particles in Hemodialysis Patients: The Sevelamer Versus Calcium to Reduce Fetuin-A-Containing Calciprotein Particles in Dialysis (SCaRF) Randomized Controlled Trial. Kidney Int Rep 2020; 5(9):1432-1447.
